# AlGaInP Red LEDs with Hollow Hemispherical Polystyrene Arrays

**DOI:** 10.1038/s41598-018-19405-y

**Published:** 2018-01-17

**Authors:** Wen-Ching Cheng, Shih-Yung Huang, Yi-Jiun Chen, Chia-Sheng Wang, Hoang Yan Lin, Tzong-Ming Wu, Ray-Hua Horng

**Affiliations:** 1 Graduate Institute of Precision Engineering, National Chung Hsing University, Taichung, 402 Taiwan, R.O.C.; 2 Department of Industrial Engineering and Management, Da-Yeh University, Changhua, 515 Taiwan, R.O.C.; 3 Graduate Institute of Photonics and Optoelectronics, National Taiwan University, Taipei, 106 Taiwan, R.O.C.; 4 Department of Materials Science and Engineering, National Chung Hsing University, Taichung, 402 Taiwan, R.O.C.; 5 Institute of Electronics, National Chiao Tung University, Hsinchu, 300 Taiwan, R.O.C.

## Abstract

A hollow hemispherical polystyrene (HHPS) was fabricated to reduce total internal reflection in AlGaInP-based LEDs. At an injection current of 350 mA, the external quantum efficiencies of LED-I, LED-II, LED-III, and LED-IV are 20.92%, 24.65%, 27.28%, and 33.77% and the wall-plug efficiencies are 17.11%, 20%, 22.5%, and 27.33%, respectively. The enhanced performance is attributed to the light output power enhancement through the surface roughness, microlens-liked PS hemisphere, and scatter-liked HHPS array. In this paper, the rigorous coupled wave analysis (RCWA) numerical method was also conducted to demonstrate the HHPS array effectively enlarge the effective light cone.

## Introduction

Quaternary AlGaInP-based light-emitting diode (LED) technologies have been rapidly developed and improved because of their extensive high-performance applications in areas including automobile indicators, outdoor full-color displays, in-house lighting, and traffic signals^1–3^. Although the internal quantum efficiency of conventional AlGaInP-based LEDs has approached 100% through the lattice-matched epitaxial growth, the low light- efficiency remains the main issue limiting the performance of top emission AlGaInP-based LEDs. The light-efficiency is mainly restricted by the light-absorbing GaAs substrate and the mismatch of the refractive index between the various materials of the device structure, the encapsulating epoxy resin, and ambient air, which leads to multiple total internal reflections at the interfaces. Wafer bonding techniques have been used for the LEDs manufacture in which the light is not absorbed by the GaAs substrate. The LED epilayer can be transferred to a substrate with a mirror structure and fabricated as an n-side up or p-side up thin-film LED structure by transferring epilayers once or twice. The total internal reflection (TIR) problem leads to only few emitted photons in a narrow escape cone from the active regions; approximately 10% of the light can be extracted from the LED’s surface; approximately 90% of the light is trapped inside the LED^4^ and is transformed into heat, which drastically degrades the LED’s performance after long-term operation. Therefore, one of the most crucial issues in AlGaInP-based LED research is increasing the number of photons that escape the active region^5–9^. Several efficient methods have been proposed and demonstrated to increase light extraction efficiency (LEE), such as the use of simple random surface roughness structures^10^, photonic crystal structures^11^, graded reflective index materials^12,13^, and embedded (or self-assembled) nanostructures^14–16^. These technologies have been effectively demonstrated to increase LEE. However, despite these efforts, certain basic material issues remain to be solved. Recently, large-scale arrays of ordered polystyrene (PS) nanospheres spin-coated on AlGaInP-based LED surfaces have been proposed as a cost-effective way to improve LEE in LEDs^17–19^. In this paper, the fabrication and characterization of p-side up thin AlGaInP-based LEDs with hollow hemispherical PS (HHPS) arrays are reported. The HHPS structures were prepared through atomic layer deposition (ALD). The electrical properties of AlGaInP-based LEDs with HHPS arrays were measured. The optical characteristics of AlGaInP-based LEDs with HHPS arrays were simulated and analyzed.

## Results

### HHPS device fabrication

**Figure 1:**
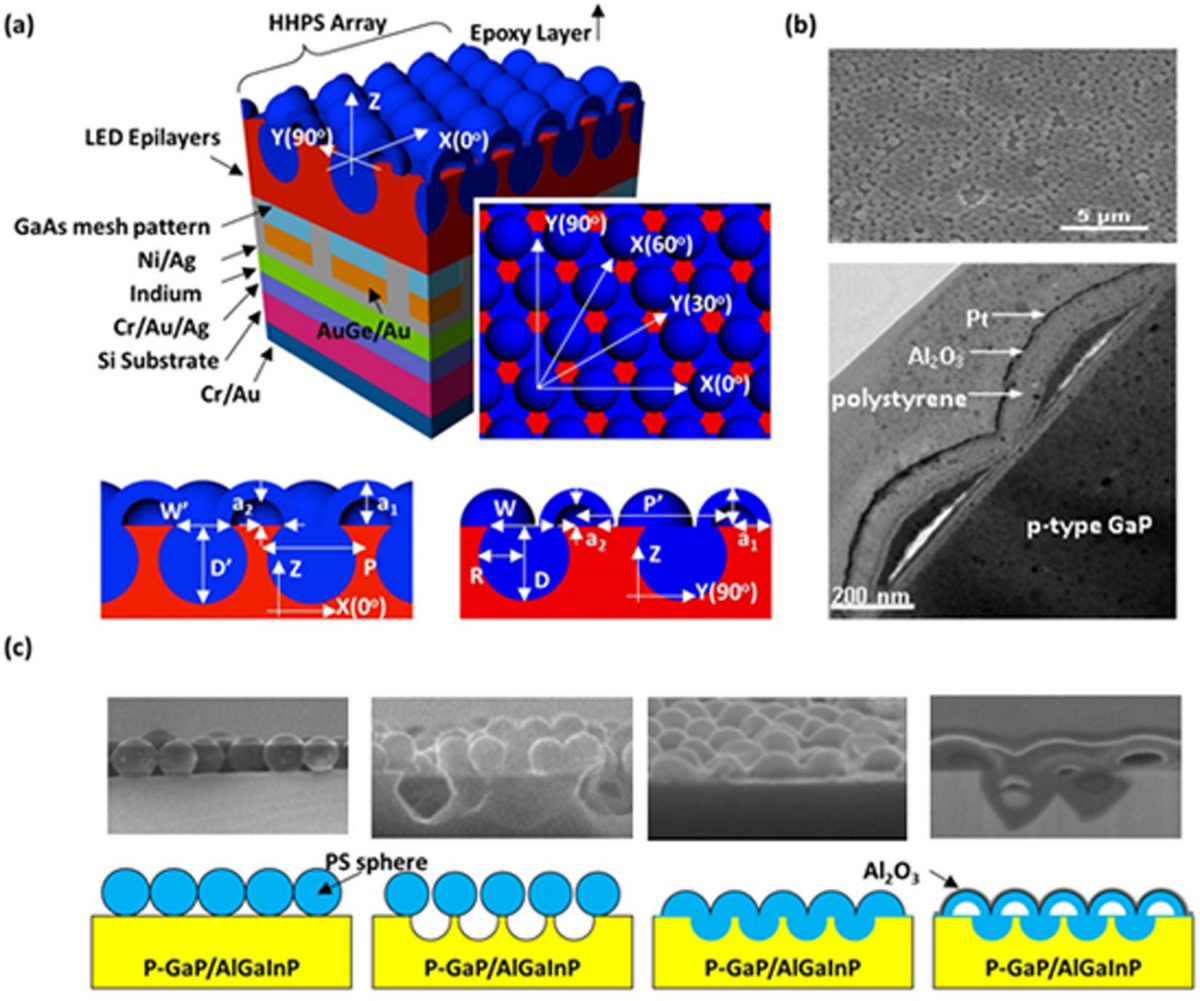
(**a**) Structural diagram of the p-side up AlGaInP-based LED with HHPS array and its Cross-sectional views in x(0^◦^) and y(90^◦^) directions. (**b**) The plane-view SEM (top) and cross-sectional view TEM (bottom) images of HHPS. (**c**) Schematic of the HHPS array fabrication process on AlGaInP-based LEDs.

LED epitaxial structures emitting light at peak wavelength (λ) about 630 nm were grown on GaAs substrates through metal organic chemical vapor deposition (MOCVD). Several p-side up AlGaInP-based thin-film LEDs were fabricated into a mesa size of 1.02 × 1.02 mm^2^ using photolithography, etching, evaporation, and wafer bonding processes. This type of LED is called LED-I. Details on the fabrication of p-side up AlGaInP-based thin-film LEDs have been reported^12^. Structural diagram of a p-side up AlGaInP-based LED with HHPS array is shown in Fig. 1(a). The plane and cross-sectional views of an HHPS array through scanning electron microscopy (top, SEM) and transmission electron microscopy (bottom, TEM), respectively, are also shown in Fig. 1(b). The fabrication process of the HHPS array is shown in Fig. 1(c).

Monolayers with arrays of 600-nm-diameter PS nanospheres were spin-coated onto the surfaces of the thin-film LEDs. The surface of top layer of GaP was roughed (with a depth of approximately 540 nm) through a natural PS lithography technology and using C_l2_/BC_l3_/Ar plasma by inductively coupled plasma reactive ion etching (ICPRIE) system. Here, LED-II denotes the type of LEDs with rough GaP surfaces. To change the PS morphology from sphere to hemisphere, the samples were dipped in a toluene solution for 15 min. LED-III denotes the type of LEDs with rough GaP surfaces and hemispherical PS coating. LED-IV denotes the type of LEDs with HHPS arrays fabricated by the following process: An Al_2_O_3_ film was deposited on the top of the array of PS hemispheres through ALD at a deposition temperature of 80 °C. The deposition temperature not only ensured the thickness uniformity of the Al_2_O_3_ film^20^, but also formed HHPS structures simultaneously (PS operating temperature ≤65 °C. Furthermore, the refraction index of Al_2_O_3_ (n ≈ 1.65) is similar to that of PS (n ≈ 1.6) at 630 nm and the Al_2_O_3_ served as a protective mask that prevented the HHPS structure from collapsing at 80 °C.

### Characterization of HHPS device

**Figure 2:**
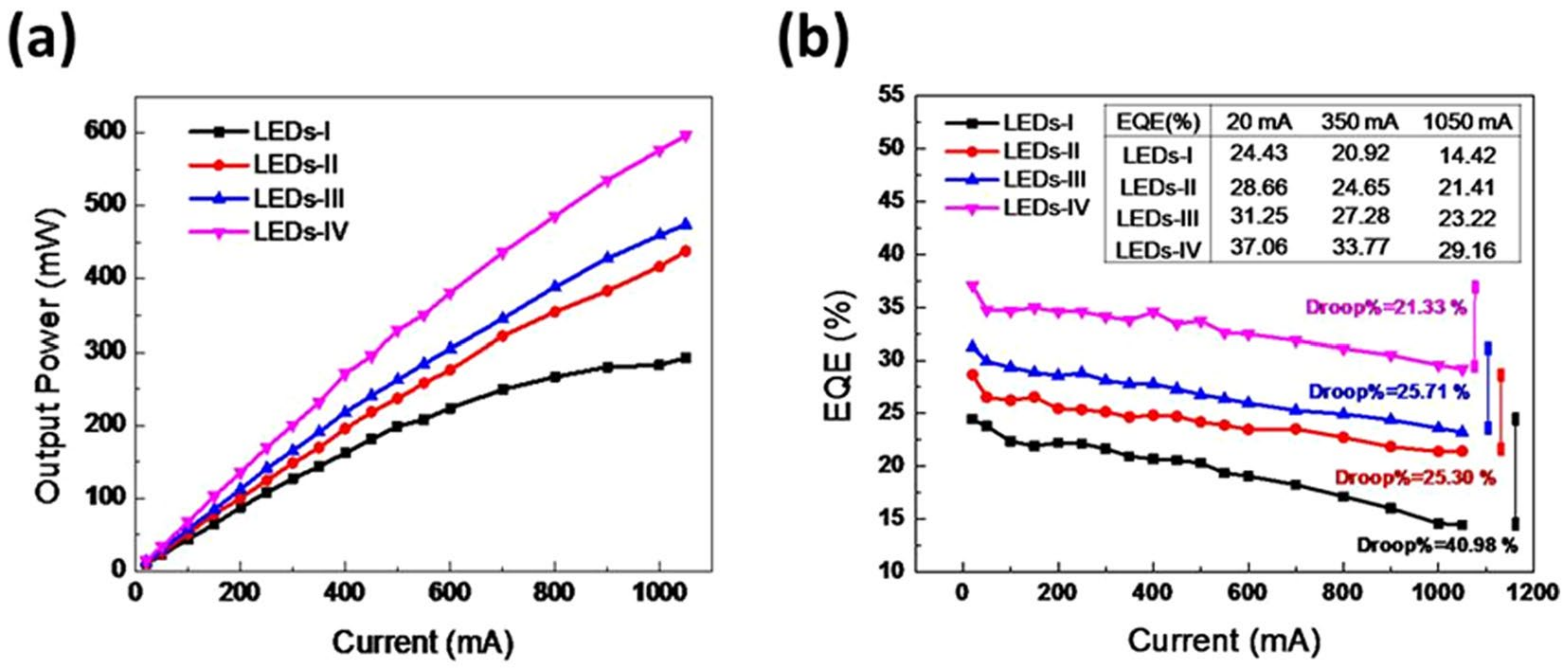
(**a**) Light output power levels against injection current for the four types of LEDs. (**b**) EQE as a function of injection current for the four types of LEDs.

Figure 2(a) shows the light output power levels of these four types of LEDs (after packaging) as functions of injection current. Under an injection current of 350 mA, the light output power levels are 143.62, 169.63, 190.70, and 231.62 mW for LED-I, LED-II, LED-III, and LED-IV, respectively. Figure 2(b) presents the EQE with respect to the forward currents of the four types of LEDs. The EQEs varied markedly upon increasing the current from 20 to 1050 mA, and all types demonstrated EQE droops with negative slopes for currents higher than 1050 mA. At an injection current of 350 mA, the EQEs of LED-I, LED-II, LED-III, and LED-IV were 20.92%, 24.65%, 27.28%, and 33.77%, respectively. When the current was increased to 1050 mA, the EQEs of LED-I, LED-II, LED-III, and LED-IV were 14.42%, 21.41%, 23.22%, and 29.16%, respectively. The efficiency droop values of LED-I, LED-II, LED-III, and LED-IV were 40.98%, 25.30%, 25.71%, and 21.33% with respect to the currents from 20 to 1050 mA, respectively. This phenomenon is due to the influence of the nonradiative recombination that increases as the injection current increases and becomes dominant at high current values^21,22^. The performances of these devices are summarized in Table 1.

**Table 1:** The summary of device performances.

	LED-I	LED-II	LED-III	LED-IV
Roughness	×	O	O	O
PS sphere	×	Sphere	Hemisphere	Hollow-Hemisphere
Light Output Power (mW) @350 mA	143.62	169.63	190.70	231.62
LOP enhancement ratio (%)	0	18.11	32.78	61.28
EQE (%) @20 mA	24.43	28.66	31.25	37.06
EQE (%) @350 mA	20.92	24.65	27.28	33.77
EQE (%) @1050 mA	14.42	21.41	23.22	29.16
Efficiency droop (%) (@1050 mA/@20 mA)	40.98	25.30	25.71	21.33
WPE (%) @350 mA	17.11	20.00	22.50	27.33

**Figure 3:**
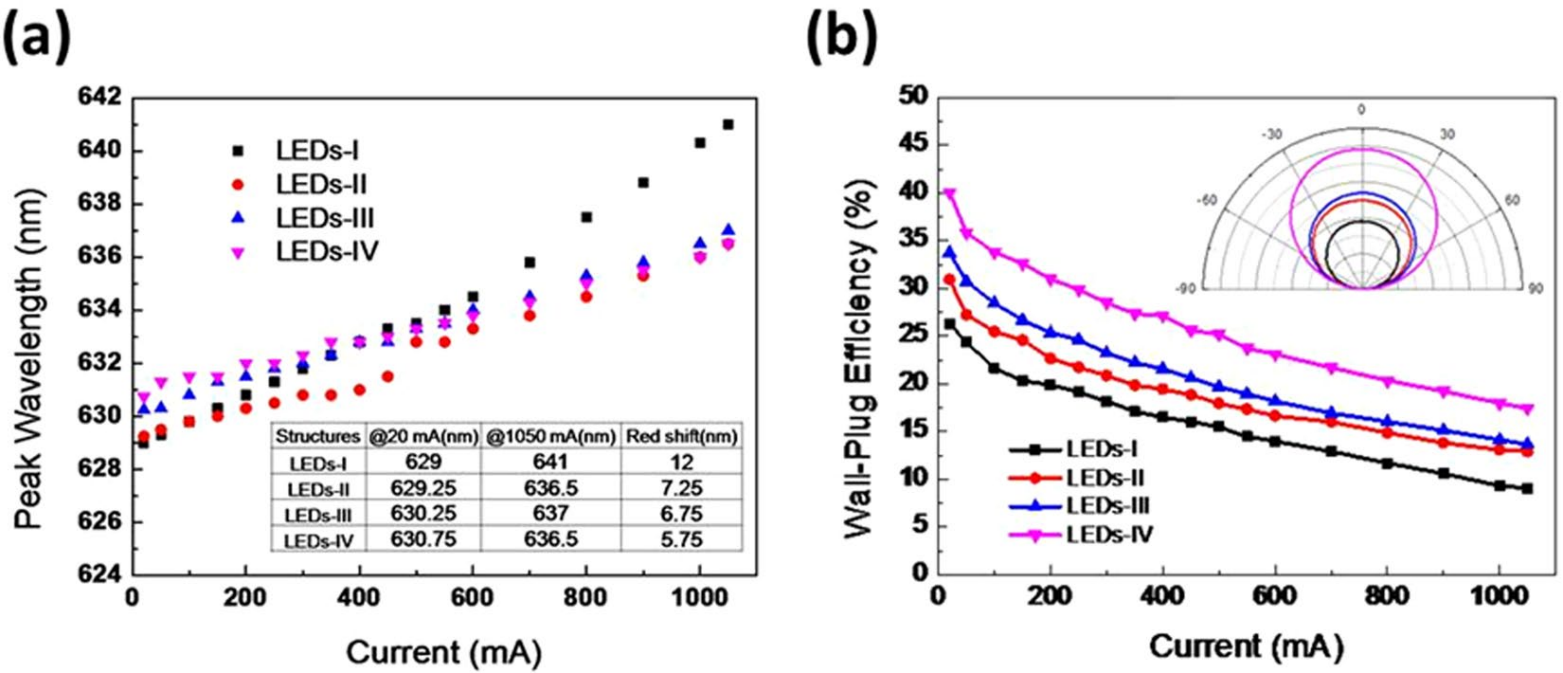
(**a**) Peak wavelength as a function of injection current for the four types of LEDs. (**b**) Wall-plug efficiency against injection current for the four types of LEDs. The angular optical distributions of the four types of LEDs at 350 mA are also shown in the inset.

The four types of LEDs demonstrated shifts in peak wavelength as a function of injection current, as shown in Fig. 3(a). It is well known that the red shift of the AlGaInP LED results from an increase in junction temperature^23^. Epitaxial growth variations cause wavelength variations at an injection current of 20 mA for all four types of LEDs (629–630.75 nm). When injection current increases, the current dependences of LED-II, LED-III, and LED-IV show the shift to longer wavelengths at rates (∆λ/∆I) of 7, 6.5, 5.6 nm/A, respectively, all of which are lower than that of the LED-I wavelength shift rate of 12 nm/A. The lower the shift to longer wavelength rate corresponds to the lower the increase of junction temperature. Therefore, it can be concluded that the superior light extraction of the HHPS array structure efficiently reduces the junction temperature and provides the optimal wavelength stability. The wall-plug efficiencies (WPEs) of the four types of LEDs as functions of injection current are shown in Fig. 3(b). The WPE values at an injection current of 350 mA for LED-I, LED-II, LED-III, and LED-IV were estimated to be 17.11%, 20%, 22.5%, and 27.33%, respectively. The enhancement rates of the WPE values of LEDs-IV were increasing approximately 60% as compared to the LEDs-I. The angular optical distributions of the four types of LEDs at 350 mA are also shown in the inset of Fig. 3(b). Compared with that of LED-I, the optical intensities of LED-II, LED-III, and LED-IV all showed obvious enhancements at 0^◦^, 30^◦^, and 60^◦^ and no obvious specular emission as expected for this scatter-used extraction method.

**Figure 4:**
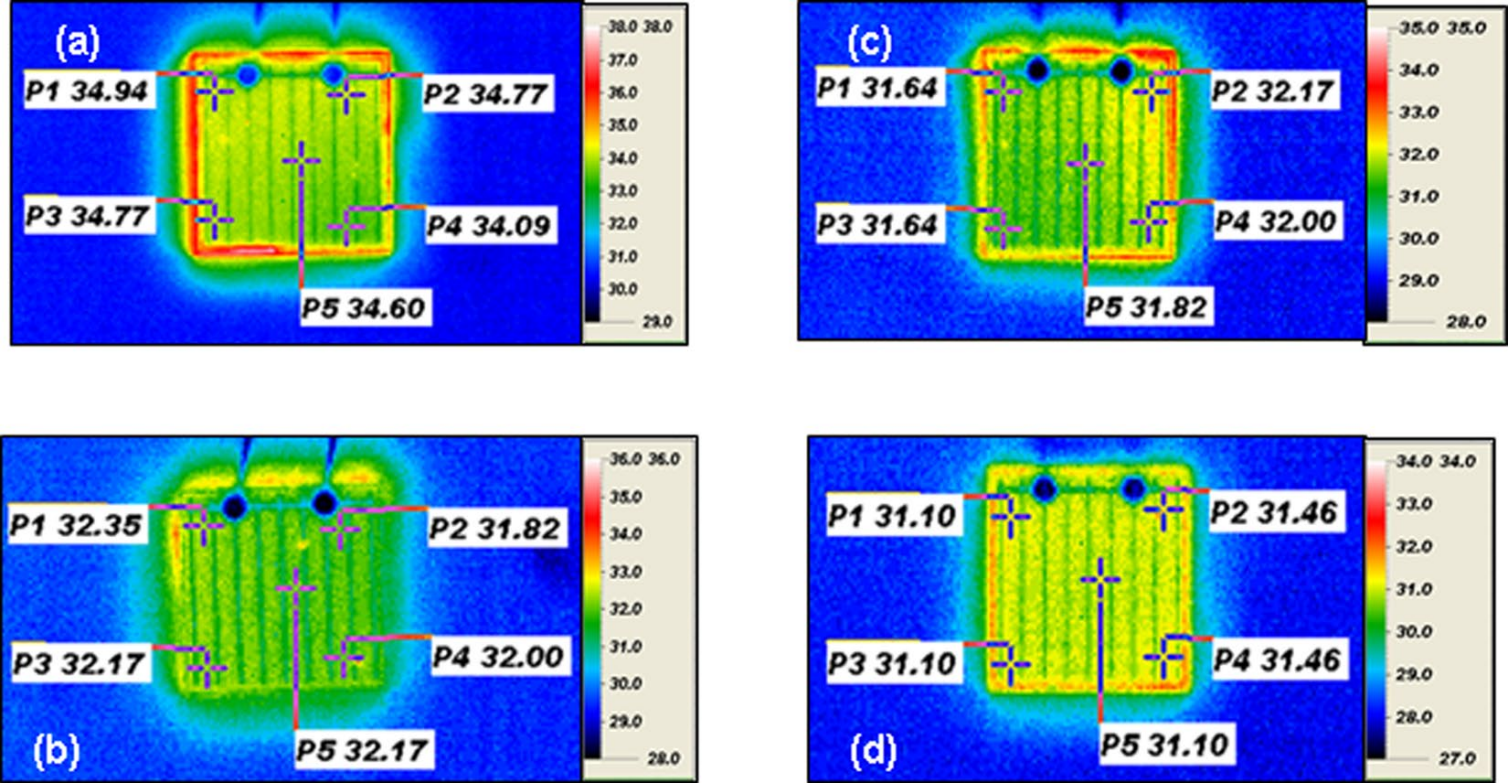
Surface temperatures of (**a**) LED-I, (**b**) LED-II, (**c**) LED-III and (**d**) LED-IV operated at 350 mA injection current.

Figure 4 shows the surface temperatures of (a) LED-I, (b) LED-II, (c) LED-III and (d) LED-IV operated at 350 mA injection current. Here, all the LEDs have the same packaged, i.e., heat dissipation rate is the same for these LEDs. It was found that the surface temperature distribution was 34.09–34.94 °C for the LED-I. Correspondingly, there were 31.82–32.35 °C, 31.64–32.17 °C, and 31.10~31.46 °C for LED-II, LED-III and LED IV, respectively. Obviously, LED-IV presented the lowest temperature. Due to the heat dissipation rate being the same for these packages, the lowest surface temperature of LED-IV resulted from the best external light extraction. The obtained results are consistent with the tendency of wavelength shift rate discussed above.

### Simulation of HHPS device

**Figure 5:**
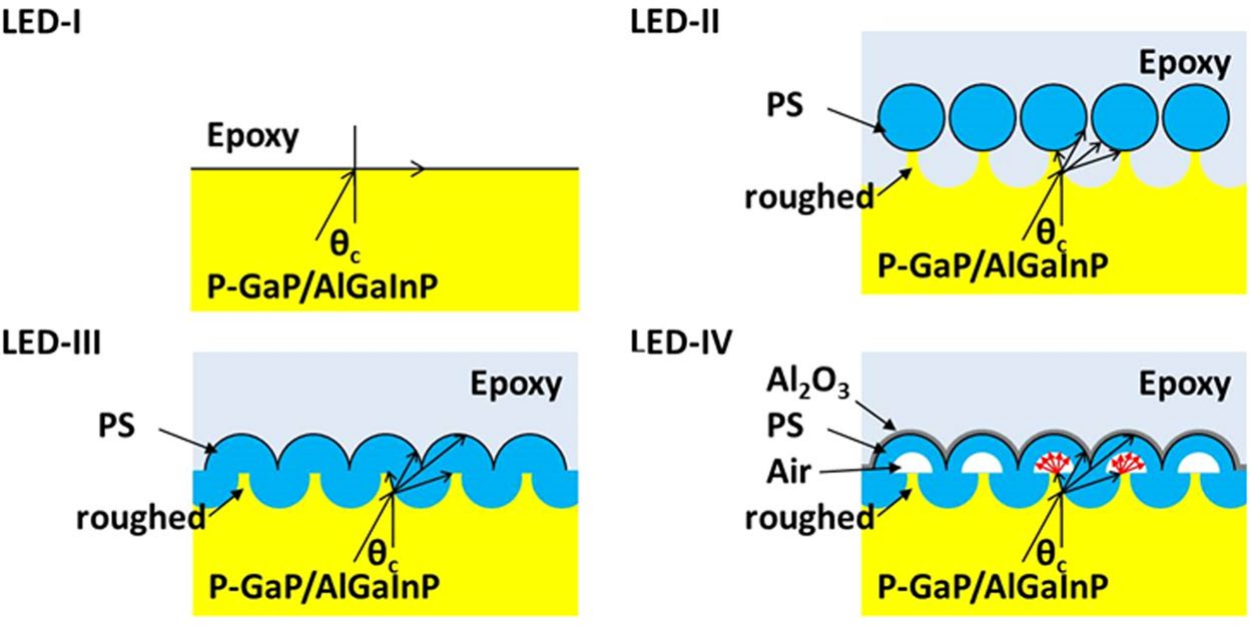
Schematics and light routes of the LED-I, LED-II, LED-III, and LED-IV.

The light output power enhancement is attributed to the larger effective light cone through the roughed surface (i.e., LED-II), the microlens-like PS hemisphere (i.e., LED-III), and the scatter-like hollow (air-embedded) PS hemisphere (i.e., LED-IV). The light routes of the LED-I, LED-II, LED-III and LED-IV were shown in Fig. 5. The large refractive index difference between the GaP window layer (n ≈ 3.5) and the epoxy layer (n ≈ 1.5) restricts the effective light cone within θ_c_ ≈ 25.4^◦^. In order to extract more light from the LED epilayer, the roughed surface was introduced by using natural PS lithography and followed by microlens-like PS hemisphere^24^. The roughed surface makes the microlens-like PS hemisphere work effectively in spite of the large index difference between the GaP window layer and the PS hemisphere (n ≈ 1.6). The roughed surface provides an effective way to reduce the TIR and results in 18.11% output power enhancement, at the same time, a 32.78% output power enhancement is demonstrated with the microlens-like PS hemisphere. Moreover, the noticeable output power enhancement to 61.28% was achieved by the HHPS array. The embedded air hole in the PS hemisphere behaves as a low index scatter, which increases the probability of light scattering and enlarges the effective light cone.

Since the scatter light routes is too complex to be demonstrated by simple ray schematics, the effective light cone is calculated by using the rigorous coupled wave analysis (RCWA) numerical method. The related geometric parameters of the simulated LED-IV model are tabulated in Table 2. Note that the shape of the roughness in the simulated model is assumed as a truncated sphere with the base diameter W, radius R, and depth D. P and P’ are the periods correspond to the X(0^◦^) and Y(90^◦^) directions. The radii of the microlens-like PS hemisphere and the embedded air-hole are labeled as a_1_ and a_2_.

**Table 2:** Geometric parameters of HHPS array in the optical model. (unit: nm).

R	P	P’	D	W	a_1_	a_2_
630	630	2Psin 60^o^	530	[(P/2)^2^ − (D − P/2)^2^]^0.5^	275	160

**Figure 6:**
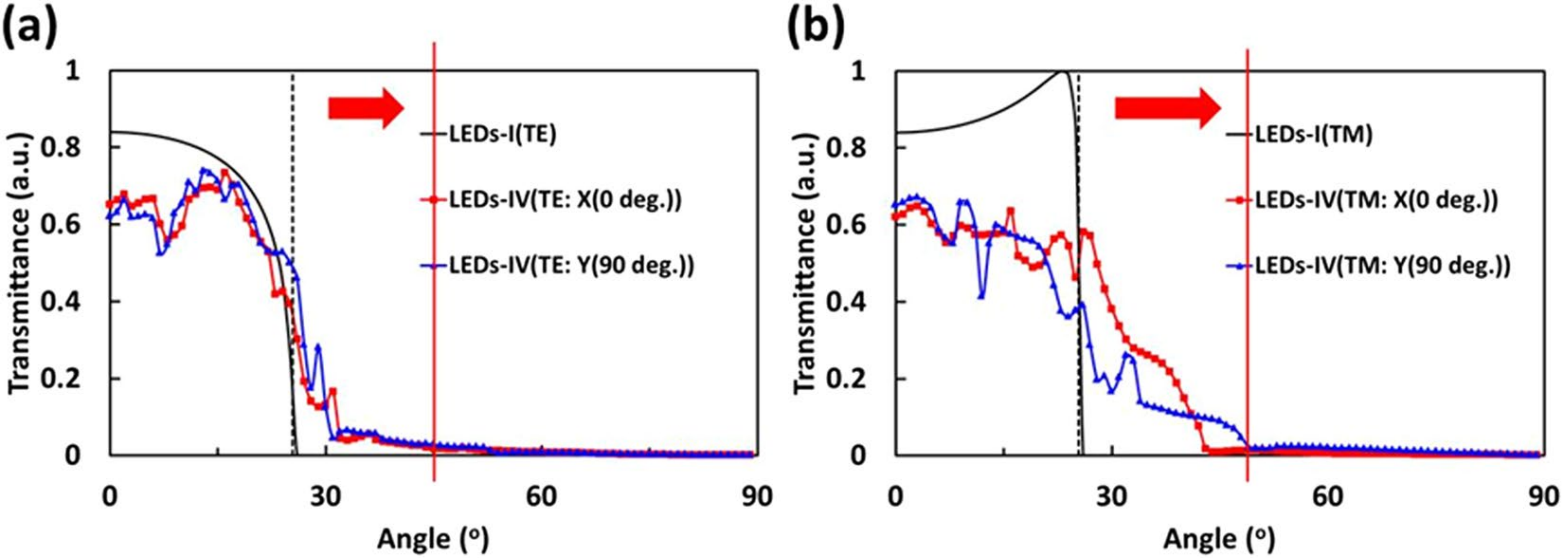
Transmittance as a function of incident angle θ_c_ taken along the X(0^◦^) and Y(90^◦^) incidence planes: (**a**) TE polarization and (**b**) TM polarization. (black dashed line: θ_c_ ≈ 25.4^◦^).

Considering the plane wave source inside the GaP layer and emits toward to the epoxy layer with different directions, the transmittance levels of LED-I and LED-IV are calculated as functions of incident angles for different polarization conditions. As the transmittance levels shown in Fig. 6(a) and Fig. 6(b), the amount of trapped light in LED-I(≤θ_c_ ≈ 25.4^◦^), i.e., the black dashed line) that can be extracted through the HHPS array now up to 45^◦^ for TE mode. Moreover, the trapped light in LED-IV can be extended up to 45^◦^ and 50^◦^ at x- and y-axials (X(0^◦^) and Y(90^◦^) for TM mode.

**Figure 7:**
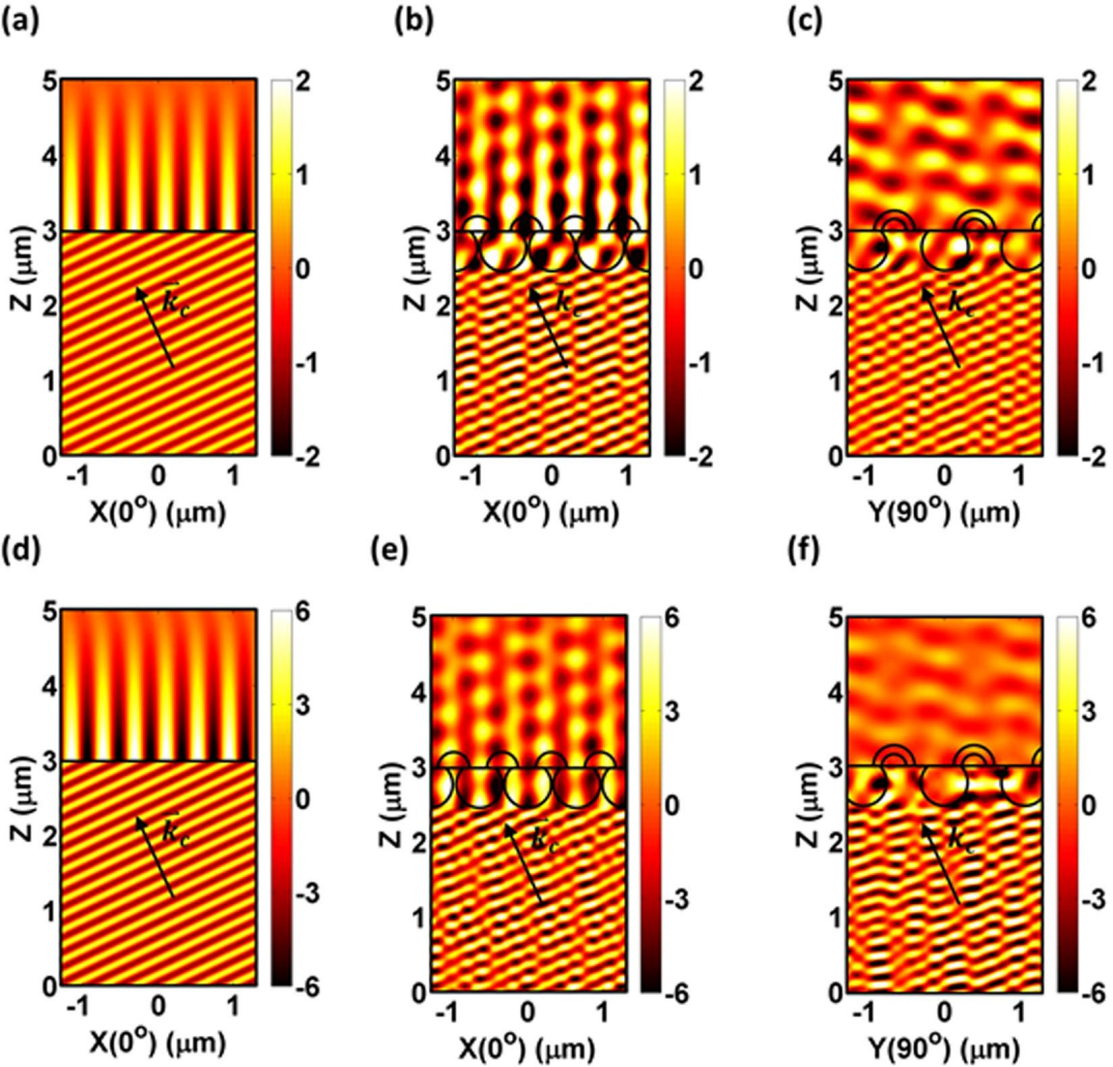
Electric field and magnetic field distributions of LED-I and LED-IV along different directions (as defined in Fig. 1 (a)) with a plane wave incidence at the critical angle (k: wave vector at θ_c_ ≈ 25.4^◦^) with respect to the z-axis). TE polarization: (**a**) LED-I [x(0^◦^)]; (**b**) LED-IV [x(0^◦^)]; (**c**) LED-IV [y(90^◦^)]; and TM polarization: (**d**) LED-I [x(0^◦^)]; (**e**) LED-IV [x(0^◦^)]; (**f**) LED-IV [y(90^◦^)].

Figure 7(a) to (f) show the electric field distributions of LED-I and LED-IV taken along different directions (i.e., X(0^◦^) and Y(90^◦^) as defined in Fig. 1(a)) with plane waves incident at the critical angles (k: wave vector with θ_c_ ≈ 25.4^◦^ respect to the z-axis). For TE polarization, an electric field in the y-direction is demonstrated, in other hand, the TM polarization was demonstrated with a magnetic field distribution in the y-direction. Clearly, the TIR occurred in LED-I, which shows that the electric field propagated along the interface and was trapped in the device (i.e., Fig. 7(a) and (d)). Furthermore, in LED-IV, the guided field penetrates into HHPS array and be coupled into the epoxy layer (i.e., Fig. 7(b),(c),(e) and (f)). Therefore, the guided field along the interface between the epoxy and GaP window layer due to the TIR (i.e., LED-I) was evidently reduced, which provides a larger effective light cone to allow more light coupling out of the LEDs.

## Discussion

The enhancement of light extraction on AlGaInP-based LEDs through the HHPS array structures was being experimentally and numerically investigated. In our experiment results, at an injection current of 350 mA, the LED-IV improved light output power levels by 61.28% compared to that of LED-I and has the less efficiency droop and peak wavelength shift as injection current varies from 20 mA to 1020 mA. In our simulations, the improved overall extraction efficiency is attributed to (approximately 1.6-fold) the embedded scatter (air hole) in HHPS array, which increases the probability of light scattering and enlarges the effective light cone. Therefore, the extraction efficiency of AlGaInP-based LEDs can be substantially enhanced, and this method provides a very promising result for the next generation of LEDs.

## Methods

LED epitaxial structures were grown on GaAs substrates by MOCVD. Twice wafer-transfer technology was used to fabricate the thin-film p-side up AlGaInP LEDs. The LED was first bonded to temporary substrate. Then, the GaAs substrate of LED was removed by solution of NH_4_OH and H_2_O_2_ (1:9). The n^+^-GaAs contact layer is protected by the GaInP as an etching-stop layer and removed by solution of H_3_PO_4_:HCl (1:2) solution. Metal films of AuGe/Au (40/60 nm) as rear-contact were deposited on n^+^-GaAs layer and annealed at 370 °C for Ohmic contact. Following, the thick metal films of silver (Ag) was deposited as the mirror and bonding layer. The metal films of Cr/Au/In were deposited on silicon substrate and bonded to LED structure with temporary glass. After bonding, the silicon substrate is thinned to 200 μm-thickness to reduce thermal accumulation of LEDs. Finally, the temporary glass substrate was removed. After the twice bonding procedure, monolayers with arrays of 600-nm-diameter PS nanospheres were spin-coated onto the surfaces of the thin-film LEDs. The surface of GaP was roughed by ICPRIE using a natural PS lithography technology. In order to obtain the hemisphere, the LEDs with PS nanospheres were dipped in a toluene solution. Moreover, LEDs with HHPS arrays can be fabricated by the following process: An Al_2_O_3_ film was deposited on the top of the array of PS hemispheres through ALD at a deposition temperature of 80 °C.
